# Financial regulation in the age of the platform economy

**DOI:** 10.1057/s41261-021-00187-9

**Published:** 2021-12-30

**Authors:** Barry Eichengreen

**Affiliations:** University of California, Berkeley, USA

**Keywords:** Financial regulation, Banks, Platform business models

## Abstract

Platform businesses allow for collaboration with nontraditional partners and bring together different categories of customers, in the financial context savers and investors or lenders and borrowers, creating large, scalable networks of users. Their entry into finance promises potential benefits to consumers in the form of new products, lower prices, wider choice, and enhanced consumer experience. At the same time, their new business models and technologies potentially threaten the dominant position of traditional financial services providers and create challenges for regulators. Platform businesses can use their preferential access to customer data to skim off high-quality loans, leaving only low-quality customers for other lenders. Their ability to offer complementary nonfinancial services that cannot be supplied by FinTech start-ups and banks can make it difficult or unattractive for customers to switch to alternative providers. This danger is especially acute when BigTech firms have monopoly power in other markets that complement financial services.

## Introduction

Financial institutions and markets are currently in a phase of unusually rapid change owing to the application of new digital technologies, including big data, artificial intelligence, and machine learning.^1^ In the first decade of the twenty-first century, innovation centered on the origination and distribution of new financial products, such as the collateralized debt obligations and credit default swaps that achieved notoriety in the Global Financial Crisis. Innovation today, by comparison, encompasses not just new products but also new processes, new interactions between financial firms and clients, and new forms of collaboration. These developments have been underway for some time; their pace has accelerated as a result of the behavioral changes and business imperatives set on foot by COVID-19.^2^

One prominent change is the proliferation of platform business models. A platform business allows for collaboration with nontraditional partners and brings together different categories of customers, in the financial context savers and investors or lenders and borrowers, creating large, scalable networks of users [3–6]. It generates and assembles information on the nonfinancial activities of clients, which can then be input into loan scoring and other financial evaluation systems.

Meanwhile, banks are partnering with financial technology companies to engineer systems with which they can attract funding from new sources, such as individuals utilizing peer-to-peer (P2P) platforms, and on which they can market nontraditional as well as traditional products. They then use information gleaned from this network of clients and transactions, processed using artificial intelligence and machine learning algorithms, to further price loans and other products, generating yet additional data in a positive feedback loop. Some banks are attempting to capitalize on these opportunities by partnering with FinTechs possessing the relevant technical expertise. Other banks are coming under pressure from the competitive entry of non-banks into the financial sphere and those non-banks’ development of their own platform models.

These changes pose challenges for regulators. New forms of interaction between financial firms and their clients have implications for consumer protection—most immediately for data protection but also for other protections—against fraud, for example. New forms of collaboration between banks and financial technology companies, and the prospective entry into the financial sector of the largest technology firms—Amazon, Google, Ant, and Tencent, for example—will require regulators to re-evaluate the implications for competition. (See Crisanto, Ehrentraud and Fabian [7] on the entry of BigTechs into finance.) The growing importance of non-bank financial technology firms will require the authorities to rethink the boundaries of the financial regulatory perimeter. And there is the danger that with new products, processes and interactions become new threats to financial stability.

The paper takes a first stab at the question of what the rise of platforms and their financial technologies imply for bank and financial regulation. Section 2 is a reconnaissance of the terrain. Section 3 then reviews rationales for regulation. Section 4 is the core of the paper: It enumerates the challenges for financial regulation posed by FinTech, BigTech, and the platform economy. Section 5 concludes with a summary of findings.

## The terrain

For centuries, banks have been in the business of maturity transformation and liquidity provision. They transform retail deposits and wholesale interbank funding, which are short in term, into long-term loans. They provide payment and transaction services. They screen customers and price their services using hard information (verifiable and codeable data from tax returns, balance sheets, credit registries, and the like) but also soft information obtained through their ongoing relationship with clients (hence “relationship banking”). They provide a range of payment, transactions, and liquidity services and use information gleaned from one interaction to inform their decisions regarding others. Universal banks, which provide the entire range of financial services, take this model to an extreme.^3^

The platform economy presents both challenges and opportunities for traditional banks. Even prior to what we know as platforms, banks were collaborating with non-bank firms and providing a growing range of financial services. They were partnering, for example, with credit card companies, which provide point-of-sale technology, while issuing actual cards themselves. They were collaborating with or acquiring brokerage firms—one thinks of Bank of America’s purchase of the brokerage Merrill Lynch—in order to provide mutual fund offerings to depositors and other clients.^4^ Thus, even before the rise of platforms, banks were diversifying their offerings and using their customer networks to cross-market new products—diversification, cross-marketing, and networks being central features of the platform economy. In this sense, banks were positioning themselves to capitalize on the platform model.

At the same time, the digital revolution and rise of platforms have increased the availability of hard (verifiable and codifiable) information relative to soft information on which banks rely. In doing so, it has eroded the value of relationship banking and diminished the banks’ traditional source of competitive advantage. The digital revolution also allows hard information to be processed more efficiently, using artificial intelligence and machine learning techniques. Insofar as banks are not early adopters of these technologies, they may again find themselves at a disadvantage relative to technology firms.

The new competitors come in a number of different shapes and sizes. The most recognizable are digital banks, which provide the same services as other banks but possess no physical branches and deliver services exclusively over the Internet. There are also FinTech firms, self-standing nonbank entities that use technology to enhance the efficiency of transactions, payments, and intermediation and that deliver these services in more customer-friendly ways. Collaborations between banks and FinTechs are increasingly prevalent; in one popular arrangement, the FinTech provides the technology for making payments and extending loans but places none of its own funds at risk, while the bank provides the capital and funds the loan [10].^5^

A further distinction is between FinTech and BigTech, where BigTech firms are large multidivisional companies with well-developed networks of customers in other markets. They apply their existing networks and proprietary information technologies to the provision of financial services. An example is Ant Financial, which utilizes information on payments gleaned from the Ant Group’s Alipay platform, used by upwards of a billion people, to inform its lending decisions.

In addition to the informational advantage derived from observing their customers’ nonfinancial transactions and activities (such as Facebook posts and Amazon purchases), BigTech firms, including Amazon, Microsoft, Google, and Alibaba, operate and control cloud computing facilities on which other financial firms rely for storage and communication. They may be able to cross-subsidize financial products using their other lines of business in order to expand their market share relative to banks.^6^ Crisanto, Ehrentraud, and Fabian [7] suggest that such network effects, positive feedbacks, and cross-subsidization may permit BigTechs to quickly become large, even dominant, providers of financial services and allow them to create barriers to entry, raising potential monopoly and too-big-to-fail concerns.

At the same time, we observe partnerships between BigTechs and traditional financial firms, including banks. Examples include partnerships between Amazon and JPMorgan Chase and between Apple and Goldman Sachs. This points to the possibility of collaboration rather than rivalry and cutthroat competition, albeit with uncertain implications for the competitive environment and consumer welfare.

Finally, there is RegTech, the name for entities that provide technological solutions to ensure the compliance of banks, FinTechs and BigTechs with applicable regulations. RegTechs may also work with supervisors and regulators to help with oversight of traditional and nontraditional financial institutions [11]. Here, it is useful to further distinguish SupTech and RegTech, which are concerned with supervision and regulation, respectively.

Ehrentraud, Ocampo, and Vega [12] distinguish not entities (banks FinTechs, BigTechs, and RegTechs)—but activities (payments, lending, investment advising, etc.). Whether to lay down rules for entities or activities is one of the fundamental tensions in regulation. In terms of the financial activities of platform firms, one taxonomy would run as follows. First there is digital banking, already mentioned, which involves taking deposits, generally under the umbrella of an existing deposit insurance scheme, and delivering traditional banking services. Second is FinTech balance sheet lending, in which FinTechs use their balance sheets (their own equity capital, debt, and securitized loans) to extend credit to borrowers. In this case, the activity is similar to that undertaken by digital banks, but funding risks are different, since there is no deposit insurance to reassure debtholders and other FinTech funders. A third activity is crowdfunding (including peer-to-peer lending), which involves soliciting funds from the public for specific purposes and on-lending those funds to borrowers. In this case, there is no use of the FinTech’s own balance sheet, except for covering operational risks. Within this third activity, Ehrentraud et al. distinguish equity crowdfunding and debt crowdfunding, depending on the form of participation by the investor.

In terms of the technologies, we have already mentioned the advantages of cloud computing for storage and communication. Smartphones with 3G or higher coverage enable contactless payments and allow digital financial services to be extended to unbanked populations, including in low-income countries. Artificial intelligence employing machine learning can be used for screening potential borrowers, including person-to-person (P2P) borrowing and lending, and for financial (robo) advising.

How widely these techniques can be applied to financial services is contested. For example, AI can be used to devise model portfolios for individuals with a given expected life span and degree of risk tolerance, enhancing recommendations by iteratively adjusting the model portfolio’s parameters based on large data inputs. But it cannot structure advice using the self-awareness, empathy, and common sense that inform the recommendations of real-life financial advisors [5]. A company like PeopleFund can develop automated proprietary credit models to screen potential borrowers and determine interest charges, but those models, unlike loan officers, cannot look those borrowers in the eye [13]. Time will tell how effectively algorithms can substitute for flesh-and-blood financial advisors and loan officers.

There is also much discussion of digital or e-currencies, although there are doubts about whether private-label digital currencies will be given regulatory authorization and whether they can compete with central bank digital currencies.^7^ Similarly, there is much discussion of blockchain and other distributed ledger technologies. These are likely to be useful for custodial functions (for attaching permanent unique identifiers to securities and other financial assets), although whether there is scope for wider use is uncertain.

Finally, there are application programming interfaces, or APIs. These are protocols specifying how different pieces of software interact; they allow multiple systems and organizations to share data and analytics. APIs allow for faster payments and easier unbundling of services. They are mechanisms for sharing data in so-called open banking applications, through which third parties can access bank data with the consumers’ consent (more on which below).

## Rationales for regulation

This proliferation of technological and organizational forms raises questions for regulators. Digital financial innovations can have benefits, from improving customer experience to enhancing financial inclusion. How then should regulators balance those benefits against risks to consumer protection, market integrity (adequate competition), and systemic stability? How worried should regulators be about stifling innovation when carrying out their prudential functions?

Regulators should start by recognizing that greater diversity of business models and organizational forms can make for a richer and potentially safer financial ecosystem. If banks fail, there still will be FinTechs; if FinTechs fail, there are also BigTechs. In this sense, the diversity of technological and organizational forms is a source of built-in redundancy and stability. The COVID-19 recession reminds us that resiliency is an important public good.

But new technological and organizational forms also create risks that will be hidden from regulatory view. They raise the danger of regulatory arbitrage if traditional banking functions are assumed by nonbank entities that operate outside the regulatory perimeter. The entry of new competition places pressure on margins of incumbent financial firms, encouraging them to gamble in order to survive. Should regulators therefore tighten rules mainly on the incumbents or entrants? These are only a few of the larger class of regulatory dilemmas.

In pondering these questions, the authorities should start by recalling the fundamental rationales for financial regulation. Conventional analyses distinguish consumer protection, market integrity, and systemic stability (see e.g., [15]). Consumers can be imperfectly informed about financial products and services, which tend to be complex and opaque. Regulation sets standards for such products and monitors the compliance of their providers in order to prevent consumers from being exploited.^8^

Market integrity refers to the maintenance of a level playing field for financial services providers and to measures intended to prevent outcomes from being manipulated by participants with market power. History is replete with instances where large investors sought to corner markets in financial assets, extracting surplus from other market participants by virtue of their size. Regulation should prevent market manipulation; it should ensure fair competition, in other words.

Systemic stability means preventing the failure of systemically important financial institutions, the collapse of markets, and other crisis-like events that threaten to disrupt the operation of the financial system. Systemic stability is fragile owing to a combination of information asymmetries, leverage, and network effects and because of the externalities to which they give rise [16]. Here, regulation can play a role ex ante and ex post, ex ante by preventing failure-prone situations from arising in the first place and ex post by facilitating efficient resolution.

But regulators, when applying rules and mandates designed to protect the consumer, ensure the integrity of the market, and maintain financial stability, must balance those benefits against compliance and other costs. The latter may take the form of static costs, but also costs associated with obstacles to innovation.

Finally, there is the choice of regulatory instrument or modality. One mode is regulation through the application of externally imposed, prescriptive, detailed rules about what is permitted and prohibited. If those rules have costly consequences, firms will have an incentive to modify their behavior so as to evade them, and the intent of regulation will be frustrated. Alternatively, regulators can attempt to design incentives compatible with the regulated firm’s own interests (they can create an incentive-compatible contract). This will encourage the regulated entity to behave in a manner consistent with the objectives of systemic stability, market integrity, and consumer protection (see [17]. This second alternative has obvious advantages, but it begs the question of whether incentive-compatible contracts can in fact be devised for the platform economy.

## Regulatory issues

With this preamble, I turn now to issues for regulation raised by the rise of financial platforms and digital finance.

### Regulatory perimeter

The advent of new organizational forms and activities requires extending the regulatory perimeter so that rules and oversight apply wherever issues of consumer protection, market integrity, and systemic stability arise. It raises the question of to what agency to assign regulatory responsibility. And it heightens the danger that activity may shift from regulated entities inside the boundary to unrelated entities outside [18, 19].

World Bank [20] notes that civil and common law countries take different approaches to extending the regulatory perimeter. Common law countries are able to utilize existing legislation, procedures, and agencies when regulating FinTechs, relying on administrative orders and decisions to accommodate new entities and activities. Civil law countries, in contrast, have had to pass new legislation in order to license and oversee FinTech entities and activities, ensuring that they are inside the regulatory perimeter. A consequent problem facing such civil law countries is that the need for detailed legislation may not keep pace with rapidly evolving financial technology practice.

Digital banks clearly should be inside the perimeter, since they differ from regulated banks only by the absence of brick and mortar. FinTech balance sheet lenders also should be inside the perimeter, since they differ from investment banks and other nonbank lenders that do not take deposits and engage in general- or specific-purpose lending only by the absence of brick and mortar. The same agency that regulates commercial banks is the logical agency to regulate digital banks, since that agency possesses relevant expertise, and since commercial and digital banks are close substitutes, which is relevant for assessing competition and market integrity. For analogous reasons, the same government agency that regulates investment banks and nonbank lenders is the obvious candidate for regulating FinTech balance sheet lenders.

OECD [21] argues for “being more lenient” toward FinTech activities that do not present systemic risks. Conceivably, crowdfunding falls under this heading, since it involves no maturity transformation and because the platform is not a liquidity provider. Crowdfunding most closely resembles mutual fund investing, where the small investor relies on a fund management company to allocate his investment to a specified set of assets and activities. Mutual funds are not subject to capital or liquidity requirements, but are required to place their assets with a qualified custodian, generally a commercial bank meeting the necessary capital requirements or with a registered broker-dealer. However, mutual funds are required to take steps to mitigate operational risks and ensure business continuity, so that investors maintain access to their accounts and transaction data; those steps may include holding capital sufficient to cover early-stage losses. Mutual funds are also regulated on consumer protection grounds—they are required to follow truth in advertising rules, for instance. Logic suggests that the same should be true of crowdfunding platforms.

In addition, because crowdfunding involves lending and borrowing by nontraditional entities, it is subject to anti-money laundering and anti-terrorist financing rules that apply to traditional financial institutions. Ehrentraud, Ocampo, and Vega [12] note that most crowdfunding platforms are subject to dedicated regulatory frameworks. (They are not regulated in exactly the same way as mutual funds or by the same regulator.) Jurisdictions also differ depending on whether they require crowdfunding platforms to hold capital and purchase professional liability insurance.

Countries differ in their approaches to licensing and regulating FinTechs that specialize in providing technology to other financial firms, including RegTechs. The European Banking Authority regards them as creating no threats to consumer protection, market integrity, and financial stability and therefore places them outside the regulatory perimeter, while China requires them to register and subjects them to regulatory oversight.

A specific issue here is what to do about BigTechs, made up of the financial divisions of large digital platform companies. A possible model is to follow the regulation of industrial banks and loan companies in the USA. Industrial loan companies are financial institutions owned by nonfinancial firms that provide niche financial services, often related to the nonfinancial firm’s core business. Examples include financial companies owned and operated by motor vehicle producers, which provide loans to car buyers via auto dealers, as well as providing real estate and related loans for car dealerships.^9^ Industrial banks and loan companies are not subject to consolidated supervision by the Board of Governors of the Federal Reserve System (the framework governing the regulation of large financial institutions). However, at the end of 2020 the Federal Deposit Insurance Corporation approved a rule requiring the parent company to conclude written agreements with the FDIC, since industrial loan companies receive deposit insurance. These written agreements include “commitments intended to protect the safety and soundness of the industrial bank and provide the FDIC with information similar to that which would be provided if the Covered Company were subject to consolidated supervision by the Federal Reserve” [22].

In principle, the obligations contained in such written agreements should allow regulators to examine whether the consolidated portfolio of the industrial loan company and its parent creates concentration or other risk. This suggests requiring such written agreements between BigTechs and the competent regulators.

However, the worry remains that lack of transparency on the part of the parent firm may hide underlying risks from regulatory view. The worry is heightened when the parent is a BigTech involved in a range of different businesses.^10^ These observations inform the view that BigTechs cannot be regulated adequately purely on a financial activity basis (that their lending activities should be regulated the same as those of other lenders, their payments services the same as those of other service providers, etc.). Rather, what BigTechs do outside the financial sphere may have implications for what they do inside that sphere, requiring them to be regulated on an entity rather than an activity basis [24].

Most countries have only begun to ponder these questions, much less respond. An exception is China, where regulators have taken strong steps to limit the non-payment-related financial business of BigTech firms, in some cases requiring them to reorganize as financial holding companies supervised by the central bank (the relevant regulator).^11^ In other words, they have seen the need to regulate them on an entity and not merely an activity basis.

### Entry regulation

Entry regulation should strike a balance between fostering innovation and competition by keeping entry barriers low on the one hand, and maintaining security and stability by requiring and enforcing specific entry requirements on the other. This dilemma is acute in the case of FinTech, the financial sphere where innovation is most rapid but also where implications for security and stability are least clear cut. It is impossible to conclude, in general, whether entry requirements should be more or less demanding than those for traditional financial institutions and activities. At a minimum, the decision of whether to issue a license for a FinTech should require evidence of adequate governance (competent and experienced management), adequate equity funding (in order to avoid excessive leverage), resources sufficient to cover early-stage losses (capital, in other words), adequate internal controls, and efficient risk management arrangements (including strong cybersecurity processes).

### Consumer protection

Regulation of FinTechs should address generic consumer protection issues but also issues specific to digital finance. Starting with the general, regulators are responsible for protecting consumers from exploitative practices. Consumers may be unfamiliar with the novel products offered by FinTech companies and therefore be vulnerable to loss-leader and bait-and-switch tactics. FinTechs may attract customers by offering low headline loan rates with additional hidden, restrictive conditions. Regulators should therefore require a minimum acceptable degree of transparency. Regulators should require FinTechs to inform customers when a financial service is still in test operation, creating unexpected risks. BigTechs with the capacity to harvest and deploy data on customer preferences and behaviors from their own platforms may have an enhanced ability to target consumers’ behavioral biases. Regulators therefore have a valid concern with platform financial companies that exploit these biases, leading investors to take on excessive risk or borrowers to take on excessive debt.

Another issue is how much control consumers have over their data. Practices vary: consumers have stronger control of their data in the European Union than the USA, for example. In the EU, banks are required to grant third-party providers access to their clients’ accounts if clients so instruct, whereas banks in the USA can deny access or charge for it [26].

The danger is that consumer-oriented data protection regimes that place demands and constraints on financial firms may render a country’s FinTechs uncompetitive internationally [20]. International agreement on minimally acceptable practices and standards is one way of addressing this problem. In 2019, the Japanese Presidency of the Group of Twenty advanced the idea of global standards for the protection, storage, and exchange of consumers’ digital data.

Regulators must also be attentive to data breaches that impose costs on consumers. Data breaches that allow third parties to access personal account information are also a problem for traditional banks. But they are likely to be an even greater problem when the financial company is part of a platform that possesses nonfinancial as well as financial data about the customer, since in this case the consumer’s purchase, medical, and other data, and not just her financial data, may be at risk. This points again to the idea that regulators should require especially strong cybersecurity processes of FinTechs. They should also require that transactions are traceable so that responsibility and liability can be assigned in the event of a security breach.

### Bias and discrimination

Many countries have laws and regulations intended to prevent providers of financial products, including lenders, from discriminating against consumers based on their race, gender, and ethnicity of religion. The challenge for regulators is distinguishing price discrimination based on such group characteristics from price discrimination based on risk.

Proprietary models and algorithms are said to be unbiased if their outputs have zero correlation with ethnic or other group characteristics once those outputs are conditioned on measures of fundamental creditworthiness [27]. The question is which measures, and whether those measures include those self-same group characteristics. Regulators address this by requiring lenders to provide a list of variables on which lending decisions are made, so as to determine whether it includes prohibited group characteristics, along with the weight attached to each variable. But as AI-based algorithms replace loan officers, the list and weights will be continuously changing with the arrival of new data points. Whether the traditional approach to determining the existence of discrimination can carry over is an open question.

In the case of algorithmic processes, moreover, the location of bias can vary [28]. The data used to “train” the algorithm may be biased. Alternatively, the training itself may be biased. An algorithm may be unbiased initially but “learn” bias from users of the platform on which it is deployed and from which it obtains data [29].

### Financial stability

Regulators concerned with the financial stability implications of FinTech can usefully distinguish two questions. First, does the model contain incentives for excessive risk-taking and inadequate risk management? Second, does such risk-taking threaten the stability of the financial system or only individual firms?

FinTech and BigTech firms specializing in technology rather than finance may lack experience and expertise in financial risk management. In any case, when such platforms simply provide technology to others, they retain no stake in the loans they help to originate. Consequently, their incentive may be to maximize loan volume and fee revenue rather than to balance revenue against risk when calibrating models and developing algorithms.^12^ As Crisanto, Ehrentraud, and Fabian (11, p.7) put it, FinTech and BigTech technology partners may have only weak incentives “for screening an monitoring clients and activities…[that could] generate excessive risk-taking behavior that could impact the financial condition or reputation of the financial firms involved.” Meanwhile, their bank partners, whose balance sheets are at risk, have little if any ability to look inside the black box of the FinTech/BigTech algorithmic process and see how risk is actually treated.

This moral hazard concern lies behind the decision of the Chinese authorities to require FinTechs such as Ant Financial to have skin in the game. In 2020, the Chinese Banking and Insurance Regulatory Commission ruled that Internet platforms had to use their own balance sheets to fund at least 30 percent of any loan extended via their co-lending partnerships with banks, whereas previously Ant Financial, the leader in this space, had funded at most 12 percent of such loans [30]. Ant being a very large FinTech co-lending with, among others, some of China’s large banks, this was an instance where moral hazard introduced by the rise of FinTech raised systemic stability concerns.^13^

This issue of whether risk is adequately priced arises as well in other contexts. In P2P lending, for example, problems of adverse selection may be accentuated by the fact that the soft information that traditional banks acquire via their relationships with borrowers is missing [13]. Consistent with this idea, smaller, younger, riskier firms possessing less collateral tend to apply for loans to platforms rather than banks, potentially heightening the risk of loan losses for the funders [31].

P2P platforms can be encouraged to attend to this problem by requiring them to co-fund their loans, like Ant is now required to co-fund the loans it helps to originate in partnership with banks. This requires them to raise equity capital sufficient for co-participation and for absorbing losses. World Bank [20] notes that capital requirements for P2P platforms vary widely. In advanced countries, unlike China, these requirements tend to be low or nonexistent. The USA, for example, has no capital requirements for P2P platforms, whereas the UK requires them to hold 0.2 percent of the total value of loaned funds. In South Korea, the Online Investment-Linked Finance and Protection of Users Act (the P2P Act) requires P2P companies to register with the Financial Supervisory Service and maintain a minimum of 500 million won (roughly $450,000) in capital.

Then, there is the threat to stability from a cyber-attack or other interruption in the operation of an electronic payments system. Banks increasingly rely on critical third-party services (e.g., data storage, transmission, and analytics), often from a single or handful of sources [7]. FinTechs as well as traditional banks rely on cloud computing, and the small handful of dominant cloud computing systems represents a rich target for hackers, terrorists, and other trouble-makers [20]. Cloud outsourcing creates operational risks for FinTechs but also risks to the stability of the financial system as a whole. To address these risks, the European Banking Authority has published guidelines for cloud outsourcing. Other financial authorities could usefully follow suit.

Finally, it is worth pondering how platforms, AI, and algorithm-based financial services will affect macro-financial volatility. Could credit and fund flows become more procyclical and unstable? Financial Stability Board [12] flags this possibility, suggesting that retail investors on P2P platforms may be more prone to panic and herd than traditional bank lenders, since the latter have past experience with credit cycles and reason to think that the central bank and regulatory authorities will stand behind them. FinTechs extending credit on the basis of algorithm-based models may all converge on similar algorithmic lending rules, leading all investors to line up on one side of the market—to all exit at the same time, for example. Kirilenko and Lo [32] speculate that algorithmic decision-making will introduce even more volatility than human decision-making. They conjecture that if some algorithm-based loans go bad, the availability of other loans will dry up, and there will be even more scope for the contagious spread of crises than under human decision-making. Algorithmic trading in securities markets has arguably had this effect; witness the risk of the “flash crash.”

The fact is that we have no idea about whether less reliance on relationships and human decision-making and more reliance on platforms, AI, and algorithmic decision-making will be stabilizing or destabilizing for the financial system as a whole. The high costs of financial crises suggest assuming initially that they will be destabilizing and therefore building stronger buffers, while standing ready to relax these if evidence accumulates to the contrary.

### Competition

At the most basic level, the advent of FinTech, by creating additional avenues for entering the financial services industry, should increase contestability and enhance competition [20]. Banks, it is argued, have long had an advantage when it comes to data on their customers. From deposit accounts to credit cards, established lenders can access information about their clients and use this to sort potential borrowers. Now, however, this competitive advantage may be eroding: FinTechs are able to mobilize information from other sources, such as digital payments transactions and social media, in order to begin to compete away the incumbents’ rents. FinTechs may also have a leg up insofar as they are free of risk and compliance obligations when they enter retail banking in partnership with banks. All this suggests that FinTech is pro-competitive.

A moment’s reflection suggests that things are not so simple, especially when one considers the activities of BigTech platforms. BigTech firms have access to still larger amounts of information, which they can use to stifle competition from FinTechs and banks. They can use their superior customer data to skim off high-quality loans, leaving only low-quality customers for other lenders. Their ability to offer complementary nonfinancial services that cannot be supplied by FinTech start-ups and banks can make it difficult or unattractive for customers to switch to alternative providers. This danger is acute when BigTech firms have monopoly power in other markets and activities, that complement financial services.

Where monopoly power does not exist, moreover, it can be created. As OECD [21] puts it, “The source of the market power of BigTech platforms is a feedback loop that generates vast quantities of customer data with the activity of the platform, processes the data with AI and ML techniques, exploits network externalities, and in turn generates more activity and more data (with dynamic economies of scale, since more data lead to better algorithms and prediction capacity). This feedback loop consolidates an ecosystem with high endogenous switching costs for customers to change platforms.” BigTechs can use the information so acquired to more efficiently price discriminate among customers, increasing their rents while also making it hard for customers to turn to competitors, who lack comparable information about those borrowers’ creditworthiness. The OECD goes on to paint a scenario where the entry of BigTech companies into financial services increases competition in the short run but in which, as the BigTechs become increasingly dominant, it stifles it in the long run.^14^

The observation that BigTechs can use their nonfinancial activities to cross-subsidize their financial business and create entry barriers to financial completion—as well as giving rise to interrelated risks—is an argument for supplementing activity-based regulation with entity-based regulation [7]. As noted above, many regulators focus on activity-based regulation (“same activity—same regulation”) as a way of limiting regulatory arbitrage and insuring that nontraditional participants in an activity are inside the regulatory perimeter. These linkages between BigTech’s financial and nonfinancial activities suggest that, from the point of view of entry regulation, it is important at the same time to apply a regulatory perspective to the entire entity and to the special problems posed by their bundling of different activities.

Open banking applications are one way of addressing problems of lock-in and inadequate competition in markets with high switching costs.^15^ These require firms to open up their customer data to third-party firms when customers provide their consent. In many cases, such as Europe’s second Payments System Directive (PSD2), banks are required to provide this information free of charge. This in turn makes it easier for consumers to shift their custom from one financial services provider to another.^16^

Early open banking rules required banks to open up their customer data to third-party firms, but did not require the same of nonbanks, notably including BigTechs.^17^ Thus, PSD2 required BigTech firms to facilitate data portability only when this is “technically feasible” [33]. Australia’s Open Banking rules, in contrast, require reciprocity: They require any accredited data recipient to provide the equivalent data to other parties [34]. As yet, however, few other jurisdictions have followed.^18^

Application programming interfaces (APIs) are another approach to problems of lock-in and imperfect competition. APIs allow for interactions between different kinds of software or software–hardware combinations. Initially, FinTechs used a process known as screen scraping to obtain customer data from banks and other financial institutions. Under screen scraping, users give the FinTech their bank username and password so that the app can then “scrape” their financial information from the bank’s Web site. Screen scraping has now given way to APIs, which allow third-party providers to plug directly into the bank website and harvest data more efficiently.

APIs offer convenience to the consumer, lower cost to third parties (which are relieved of the need to scrape the data from another source, reformat it, and ingest it), and security for all concerned (assuming of course that the API itself is subject to adequate regulatory oversight). They are pro-competitive insofar as they make it easier for consumers to switch providers. But early APIs were generally deployed to insure that information possessed by financial firms (predominantly banks) was shared with potential nonbank competitors. In the age of the platform economy, it will be equally if not more important that regulators insure that information possessed by BigTechs is shared with banks.

Still, it is not clear that this is enough. BigTechs can use their platforms to generate large amounts of customer data, employ it in training their AI algorithms, and identify high-quality loans more efficiently than competitors lacking the same information. Customers may be able to move their financial data to a bank or FinTech, but what about their nonfinancial data? What about the algorithm that has been trained up using one’s data and that of other customers? Without this, digital banks and FinTechs will not be able to price and target their services as efficiently as BigTechs. Problems of lock-in and market dominance will not be overcome. Rather, overcoming them may require mandating that BigTechs create impermeable firewalls between their financial and nonfinancial businesses (evidently, the Chinese approach) or breaking up BigTechs into smaller firms (currently under active, if inconclusive, discussion in the USA).

### Sandbox regulation

By creating a testing environment known as a sandbox, regulators of FinTech services give firms an opportunity to experiment with new products and processes free of the regulatory burdens of traditional banks, while also affording regulators an opportunity to identify effective ways of safeguarding stability and encouraging innovation [35, 36]. By creating a sandbox, the authorities signal that they are committed to fostering innovation [37]. Typically, firms operating in the sandbox receive a no-enforcement letter exempting them from a specified set of regulations, something that is likely to be especially valuable for small start-up companies otherwise facing formidable compliance costs. When new entrants to financial service provision participate, they benefit from learning to interact with regulators. Since activities in the sandbox are isolated from other services, threats to the stability and integrity of the larger system are avoided.^19^

Problems with the sandbox model include striking a balance between the competing objectives of stability and innovation. Some sandboxes favor incumbent financial institutions over start-ups by permitting only the participation of licensed providers of financial services.^20^ This creates a danger that early entrants into the sandbox may be able to influence regulation in ways that deter entry by potential competitors [39]. In practice, it may not always be possible to strictly isolate transactions initiated in the sandbox from the rest of the financial system, whether because of the integrated nature of technologies and economies or because of simple regulatory failure [40].

Regulators presumably gain from sharing lessons learned in their respective national sandboxes. In 2018, the UK Financial Conduct Authority therefore proposed creating a global sandbox, though nothing came of this.^21^ Instead, authorities agreed to create a Global Financial Innovation Network (GFIN) to share national experiences regarding best practices and to advance joint policy work by linking their respective sandboxes [11]. Two-party cooperation of this sort is especially relevant for the development of cross-border technologies, since the relevant technologies will have to be compatible with several countries’ regulatory standards [41].

## Conclusion

The financial landscape is changing, as the banks that long dominated the financial sector face competition from FinTechs utilizing new technologies, adopting novel business models, and offering innovative financial services. Entry and innovation promise benefits to consumers enjoying new products, lower prices for traditional products, wider choice, and an enhanced consumer experience. At the same time, however, new business models and technologies potentially threaten the dominant position of traditional financial services providers.

The FinTech revolution poses a challenge for regulators for both reasons. Regulators must scramble to acquire the technological expertise needed to exercise effective oversight of the entrants. They also have to prevent incumbents from gambling for redemption and understand the implications of traditional banks partnering with FinTechs and doing business in new and novel ways.

Then there are the specific challenges posed by the financial services activities of the BigTech companies that are emblematic of the platform economy. These companies provide payment, delivery, social media, and even medical services, enabling them to comprehensively monitor their customers’ economic and other activities. This gives them an informational advantage that they can deploy to price discriminate, extract rents, exploit consumers’ behavioral biases, and gain a competitive advantage over other financial service providers. When BigTechs have quasi-monopolies in markets that are complementary to finance, consumers may find it hard to switch providers and therefore find themselves locked in, with significant costs. Ensuring fair financial competition may therefore require BigTechs to create firewalls between their financial and other activities. It may lead the authorities to contemplate breaking up big platform firms.

Open-banking rules requiring firms to share information are another regulatory response, but in the past such sharing requirements have been imposed on banks, not FinTechs, much less BigTechs. In the case of BigTechs, there is also the question of exactly what information is shared: obliging competitors to share financial information but not other information creates an asymmetry: Banks have to share everything, but BigTechs are required to share only a portion of what they know. A level playing field will presumably require this to be changed.

Authorities are aware that the entry of FinTechs and especially BigTechs into the provision of financial services raises consumer protection, market integrity, and financial stability concerns. These issues are at the heart of financial regulation. Recognition is a first step in the direction of crafting a response. But it alone is not enough.

## Footnotes

1 Barry Eichengreen is George C. Pardee and Helen N. Pardee Professor of Economics and Political Science at the University of California, Berkeley, Research Associate at the National Bureau of Economic Research, and Research Fellow at the Centre for Economic Policy Research. Thanks are due to Si-Yeon Lee and John Zysman for helpful comments and to the Korea Institute of Finance for financial support. The author has no relevant conflicts of interest to report.

2 Aksoy, Eichengreen and Saka [1] show that past epidemics induced changes in banking practice away from interpersonal interaction at bank branches and toward online, Internet and cell-phone-based banking. Crosman [2] observed early in the pandemic that 35 percent of customers surveyed had increased their usage of online banking during COVID-19. The unanswered question, as Crosman notes, is whether the shift will last.

3 For the relevant history, see Fohlin [8].

4 The dominant modality remains collaboration; on this history, see Neely [9].

5 There have been some recent regulatory initiatives to require FinTechs to commit their own balance resources to lending extended in partnership with banks (see below).

6 Such cross-subsidization may be desirable insofar as customers, once they establish a relationship with a financial firm, find it hard to move their business to a competitor, effectively becoming locked in—at which point prices and profits can be raised. Open-banking protocols are one way of addressing this problem (again, see below). Carstens [2] reports data for a selection of BigTechs in 2018, indicating that finance accounts for about 10 percent of their total revenues, which suggests that the potential scope for cross-subsidization is considerable.

7 I have my doubts, which I express in Eichengreen [14].

8 To put it another way, it addresses agency problems in the financial system.

9 These include BMW Bank of North America Industrial Loan Company and the Toyota Financial Savings Bank.

10 South Korea has faced a broadly analogous problem independent of the rise of the platform economy, since many of its large industrial and commercial conglomerates (chaebol) had already branched into finance in earlier years. In 2020, it adopted a new Financial Conglomerates Supervision Law and named Samsung, Hyundai, Hanwha, Mirae Asset, Kyobo and DB (on the grounds that they had affiliates in two or more financial sectors with combined assets of 5 trillion won) as conglomerates subject to the provision, requiring them to improve their internal controls and risk management. IMF [23] recommended extending the authorities’ supervisory and regulatory powers to non-financial institutions of the wider group, but it is not clear that this has been fully accomplished by the 2020 law.

11 In an April 30, 2021, statement, the PBOC asserted that BigTech firms should “disconnect improper links between payment services and other financial products, strictly control the expansion of non-bank payment accounts to the public domain, improve transaction transparency, and correct unfair competition” [25].

12 A related risk is that BigTechs for their part may over-expand loan provision if doing so boosts their other platform business.

13 In part to address them, the Chinese Regulatory Commission also limited banks to extending no more than 50 percent of their loans through FinTechs partners and limited such lending to 25 percent of Tier 1 capital.

14 For a related discussion, see [41]. Whether the increasing dominance of vertically as well as horizontally integrated BigTech firms leaves consumers better or worse off depends, in the end, on what one imagines the alternative to be. If the alternative is a more competitive financial services industry, then the rise of BigTechs leaves consumers worse off, other things equal. On the other hand, if the alternative is a set of imperfectly competitive FinTechs providing technology to a set of imperfectly competitive banks, then there is the possibility of “double marginalization,” in which both the Fintechs and banks charge quasi-monopoly markups, leaving consumers even worse off than when there is a vertically integrated BigTech supplier.

15 There are now many other examples of open banking rules, such as the 2015 EU Payment Services Directive II (PSD2).

16 In addition, having this information feed into a single app should enhance customer experience by displaying a comprehensive list of his or her accounts across banks.

17 Ref. [21] point out that this is a continuing problem in a number of jurisdictions.

18 China is discussing whether to require BigTechs to share their credit scoring systems with the authorities and, by implication, with other FinTechs and banks, as a way of addressing the problem that debtors have tended to borrow on multiple platforms. Unlike in other cases, this particular form of open banking would work to the benefit of the banks and to the disadvantage of BigTechs.

19 South Korea launched its regulatory sandbox scheme in 2019, under the provisions of its Special Act on Financial Innovation Support, described above. Under its provisions, Fintech firms and other recognized providers of “innovative financial services” are exempted from certain regulations for a two-year, once-renewable period.

20 In China, several separate cities have launched regulatory sandboxes [38]. Incumbent banks look to be the dominant players.

21 In China, where there exist competing sandboxes at the municipal level, this problem of information sharing occurs at the national level as well.
